# CD4^+^ skin resident memory T cells preferentially colocalize with dermal Folr2^hi^ macrophages in contact hypersensitivity

**DOI:** 10.3389/fimmu.2025.1590687

**Published:** 2025-07-28

**Authors:** Akihiko Murata, Koji Tokoyoda

**Affiliations:** Division of Immunology, Department of Molecular and Cellular Biology, School of Life Science, Faculty of Medicine, Tottori University, Yonago, Tottori, Japan

**Keywords:** tissue-resident memory T cells, Folr2^hi^ macrophages, local immune memory, contact hypersensitivity, allergic contact dermatitis

## Abstract

In contact hypersensitivity (CHS), local immune memory is established in previously affected skin through the formation of CD4^+^ and CD8^+^ tissue-resident memory T (T_RM_) cells. This memory contributes to disease recurrence by enhancing local antigen responsiveness and is maintained in the long term by T_RM_ cells, particularly CD4^+^ T_RM_ cells. However, the mechanisms underlying the maintenance and reactivation of CD4^+^ T_RM_ cells remain unclear. We herein examined the cellular niches persistently interacting with CD4^+^ T cells in naïve and CHS-healed mouse ear skin. Most CD4^+^ T cells were scattered in the dermis and colocalized with Folr2^hi^ macrophages, a previously unrecognized skin macrophage population, suggesting a physical interaction. In contrast, fewer than 20% of CD4^+^ T cells colocalized with dendritic cells (DCs) or other cell lineages. The administration of an anti-colony stimulating factor 1 receptor (CSF1R) antibody depleted nearly all Folr2^hi^ macrophages and several other myeloid cells, while the maintenance and reactivation of CD4^+^ T cells as well as other αβ T cells in healed skin remained unaffected. Moreover, in macrophage-depleted healed skin, CD4^+^ T cells did not establish new interactions with remaining antigen-presenting cells, and their contact rate with DCs remained unchanged. These results indicate that local immune memory in CHS-experienced skin is maintained and functions independently of CSF1R-dependent myeloid cells, including Folr2^hi^ macrophages, despite their predominant colocalization with skin CD4^+^ T_RM_ cells.

## Introduction

Allergic contact dermatitis (ACD) is T cell-dependent skin inflammation characterized by recurrent episodes of relapse and remission at the same site (1, 2). In an experimental model of ACD, contact hypersensitivity (CHS) induced by the application of a hapten, it has been shown that in the skin of rodents where CHS has resolved, antigen re-exposure leads to the localized and antigen-specific amplification of sensitivity and the inflammatory response (3, 4). This heightened responsiveness upon antigen re-exposure persists for more than one year after the resolution of CHS in BALB/c mice (5). These findings suggest the formation of localized immune memory in the previously affected skin area. Since the identification of tissue-resident memory T cells (T_RM_ cells) (6), they have been proposed as key mediators of local skin immune memory (7).

CD4^+^ and CD8^+^ T_RM_ cells are subsets of memory T cells that are characterized by their residency in inflammation-experienced tissues and unique transcriptional profiles distinct from circulating memory T cells (8–12). In response to local cytokine signals within tissues, T_RM_ cells differentiate from infiltrating activated αβ T cells (or T_RM_ precursors) and are maintained after returning to a steady state (13). In the skin of mice, CD4^+^ and CD8^+^ T_RM_ cells are mainly found in the CD69^+^CD103^+^ compartment (14–17). CD8^+^ T_RM_ cells predominantly localize in the epidermis, while CD4^+^ T_RM_ cells are almost exclusively found in the dermis (18).

A previous study on C57BL/6 mice showed that only epidermal CD8^+^ T_RM_ cells formed in CHS-healed ear skin, which mediate enhanced antigen responsiveness (19). In CHS-experienced BALB/c mice, we recently demonstrated the following: 1) in addition to epidermal CD8^+^ T_RM_ cells, dermal CD4^+^ T_RM_ cells formed in healed ear skin, 2) the presence of CD4^+^ T_RM_ cells or CD8^+^ T_RM_ cells alone was sufficient to amplify the inflammatory response upon antigen re-exposure, and 3) epidermal CD8^+^ T_RM_ cells disappeared over time, whereas dermal CD4^+^ T_RM_ cells were maintained for more than one year. These findings indicate the existence of two independent mechanisms of local immune memory in the skin mediated by epidermal CD8^+^ T_RM_ cells and dermal CD4^+^ T_RM_ cells (20). Furthermore, the long-term persistence of CD4^+^ T_RM_ cell-mediated local immune memory may contribute to the sustained enhancement of local antigen responsiveness and play a role in the recurrence of ACD. In addition, CD4^+^ T cells, but not CD8^+^ T cells, have been shown to persist in the dermis of patients with resolved ACD (21).

Therefore, to prevent the recurrence of ACD by addressing local immune memory, it is necessary to understand the mechanisms underlying the maintenance and reactivation of T_RM_ cells. The mechanisms responsible for the maintenance and reactivation of epidermal CD8^+^ T_RM_ cells have been extensively examined. Their maintenance depends on signals, such as transforming growth factor (TGF)-β, which they produce and is transactivated by adjacent keratinocytes (22–24), as well as interleukin (IL)-7 and IL-15, which are produced by keratinocytes and Langerhans cells (LCs) with which they interact (23, 25–28). Additionally, cross-presenting LCs are essential antigen-presenting cells (APCs) for the reactivation of epidermal CD8^+^ T_RM_ cells upon viral reinfection (27). These findings suggest that keratinocytes and LCs serve as niches required for the maintenance and reactivation of epidermal CD8^+^ T_RM_ cells.

In contrast, while CD4^+^ T_RM_ cells have been shown to form in various skin infections and pathological conditions, limited information is available on their cellular niches (11, 29, 30). In mouse trunk skin, 50% of CD4^+^ T_RM_ cells induced by hapten sensitization or pathogen infection form clusters with major histocompatibility complex (MHC)-II^+^ APCs containing dendritic cells (DCs) and macrophages around hair follicles, which may be involved in the maintenance and reactivation of CD4^+^ T_RM_ cells (31, 32). The maintenance of CD4^+^ T_RM_ cells is regulated in a subset-specific manner, with IL-23-producing CD301b^+^ APCs being essential for the maintenance of Th17-type CD4^+^ T_RM_ cells (32). Moreover, accumulated DCs and macrophages are sustained in healed skin following viral infection (14). On the other hand, in a fungal infection model of mouse ear skin, CD4^+^ T_RM_ cells did not form clusters, but were instead scattered and colocalized with MHC-II^+^ APCs (15). These findings suggest that APCs, such as DCs and/or macrophages, serve as niches for the maintenance and reactivation of dermal CD4^+^ T_RM_ cells; however, it remains unclear which cell lineages and subsets are involved in CHS-experienced skin.

In the present study, we attempted to identify the cellular niches for CD4^+^ T_RM_ cells in the CHS-experienced ear skin of BALB/c mice. Specifically, we investigated the cell lineages that continue to interact with CD4^+^ T_RM_ cells and examined whether their depletion affects the maintenance and reactivation of CD4^+^ T_RM_ cells, in addition to other αβ T cell lineages in healed skin. The results obtained herein revealed that most dermal CD4^+^ T_RM_ cells persisted and closely colocalized with a previously unrecognized macrophage subset, Folr2^hi^ macrophages. However, these macrophages, as well as several types of skin myeloid cells, were not essential for the maintenance and reactivation of not only CD4^+^ T_RM_ cells, but also other αβ T cells that remained in healed skin.

## Materials and methods

### Mice

BALB/c and C57BL/6J mice (The Jackson Laboratory Japan, Kanagawa, Japan) and I-A^b^-restricted lymphocytic choriomeningitis virus (LCMV) glycoprotein (GP)_61-80_-specific T cell receptor transgenic SMARTA mice (33), kindly provided by Dr. Max Löhning, were maintained in a specific pathogen-free facility at Tottori University. All experiments were approved by and performed in strict accordance with the guidelines of the Animal Care and Use Committee of Tottori University (Approval numbers: 19-Y-51 and 22-Y-13).

### CHS

The hapten 2,4,6,-trinitrochlorobenzene (TNCB) (Tokyo Chemical Industry, Tokyo, Japan) was dissolved in vehicle (acetone:olive oil = 4:1) (FUJIFILM Wako Pure Chemical Corporation, Osaka, Japan). On day -7 or -6, the right ears of BALB/c mice were sensitized with 20 μl of 40 mM TNCB (10 μl, both sides of the ear) under isoflurane anesthesia. On day 0, the right ears were challenged with 20 μl of 40 mM TNCB, while the left ears received 20 μl of vehicle (10 μl, both sides of the ear). In some experiments, both ears were sensitized and challenged. Hapten applications and measurements were conducted between 7:00 a.m. – 2:00 p.m.

### Delayed-type hypersensitivity

Naïve splenic CD4^+^ T cells from SMARTA mice (Thy1.1^+^) were sorted with the MojoSort Mouse CD4 Naïve T Cell Isolation Kit and MojoSort Magnet (BioLegend, San Diego, CA), and were suspended in 0.1% bovine serum albumin (BSA, FUJIFILM Wako) in phosphate-buffered saline (PBS). Under isoflurane anesthesia, C57BL/6 mice (Thy1.2^+^) were adoptively transferred intravenously with 10^6^ SMARTA T cells on day -8, sensitized by the subcutaneous injection of emulsified LCMV GP_61–80_ peptide (synthesized by GeneCust, Boynes, France) plus Complete Freund’s Adjuvant (CFA, FUJIFILM Wako) (mixed 1:1) into the middle of the back and buttocks on day -7 (50 μg peptide in 50 μl/site), and challenged by an intradermal injection of LCMV GP_61-80_ (approximately 40 μg/40 μl/ear) into the dorsal side of right ears on day 0. Ear thickness was measured with the dial thickness gauge G-1A (Peacock) under isoflurane anesthesia.

### Whole mount immunostaining

After removing hair using depilatory cream, ears were excised at the base, washed in PBS, and split into dorsal and ventral halves. The dorsal ear halves without cartilage were fixed by floating with the dermal side down in 4% paraformaldehyde in PBS (PFA, FUJIFILM Wako) at 4°C for 6 h. The ears were then incubated with 20% goat serum for blocking (at 4°C overnight), followed by Streptavidin/Biotin Blocking Solution (at room temperature (RT) for 6 hours for each solution, Thermo Fisher Scientific, Waltham, MA). Ears were subsequently incubated with 20% goat serum containing anti-CD4-biotin (RM4-5, BioLegend) and MHC-II-Alexa Fluor 488 (M5/114.15.2, BioLegend) (at 4°C overnight), followed by Streptavidin-BV421 (at RT for 2.5 hours, BioLegend) in microtubes. Between each step, ears were thoroughly washed several times in 0.3% Triton X-100 in PBS. To permeabilize tissues, Triton X-100 was added to all solutions, except PFA (final concentration: 0.3% v/v).

Ears were mounted on slides in Dako Fluorescence Mounting Medium (Agilent Technologies, Glostrup, Denmark) with the dermal side facing up and in contact with the cover slip. Tiled, z-stacked images were acquired using an LSM900 confocal microscope with 10× (786×786 resolution, averaging: 2, speed: 7, 1.67 μm thickness × 6 slices) and 20× objective lenses (1024×1024 resolution, averaging: 4, speed: 7, 1.67 μm thickness × 15 slices), and processed with ZEN Microscopy software (Zeiss, Jena, Germany).

### Immunostaining of ear sections

The central region of the ears was excised and snap-frozen in OCT compound (Sakura Finetek Japan, Tokyo, Japan) using liquid nitrogen. Horizontal sections (thickness of 8 μm) were prepared from the base of the ears using a CM-1950 cryostat (Leica, Nussloch, Germany). Sections were fixed with 4% PFA at RT for 4 minutes, followed by blocking with 20% normal horse serum (Vector Laboratories, Burlingame, CA) for Tim-4 staining or normal goat serum (FUJIFILM Wako) for other staining, in Block Ace (KAC, Kyoto, Japan) at RT for at least 1 hour. If a biotinylated antibody was used, sections were additionally blocked with Streptavidin/Biotin Blocking Solution (Thermo Fisher Scientific).

Sections were then stained with combinations of the following antibodies and reagents: anti-CD4-purified/-biotin/-APC/-BV421 (RM4-5, BioLegend), MHC-II-Alexa Fluor 488 (M5/114.15.2, BioLegend), CD45-purified (30-F11, Tonbo Biosciences), CD11b-purified (M1/70, BioLegend), CD68-Alexa Fluor 488 (FA-11, BioLegend), F4/80-purified (BM8.1, BioLegend), CD11c-biotin (N418, BioLegend), Siglec-F-purified (1RNM44N, eBioScience), CD64-APC (S18017D, BioLegend), CD163-APC (S15049I, BioLegend), CD169-Alexa Fluor 647 (3D6.112, BioLegend), CD86-purified (GL1, BioLegend), CD206-Alexa Fluor 647 (C068C2, BioLegend), Folr2-purified (10/FR2, BioLegend), Thy1.1-biotin (HIS51, eBioScience), goat anti-mouse NKp46 and Tim-4 polyclonal IgG (R&D Systems, Minneapolis, MN), and Avidin-Alexa Fluor 488 (Invitrogen). Purified and biotinylated antibodies were visualized using additional staining with AffiniPure F(ab’)_2_ fragment donkey anti-rat or anti-goat IgG(H+L)-Alexa Fluor 488/-Cy3/-Alexa Fluor 647 (Jackson ImmunoResearch, West Grove, PA) and streptavidin-Brilliant Violet (BV) 421 (BioLegend)/-Alexa Fluor 488/-Alexa Fluor 546/-Alexa Fluor 647 (Thermo Fisher Scientific). Nuclei were stained with 4’,6-diamidino-2-phenylindole dihydrochloride (DAPI, DOJINDO LABORATORIES, Kumamoto, Japan) or TO-PRO-3 (Thermo Fisher Scientific). The cell membrane was stained using CellMask Plasma Membrane Stains Orange/Deep Red (Thermo Fisher Scientific). If staining included a purified rat antibody, sections were initially stained with the purified antibody and a fluorescent anti-rat secondary antibody, followed by blocking with rat serum for 30 minutes and subsequent staining with the remaining antibodies, including labeled rat antibodies. We confirmed that this procedure completely blocked the non-specific binding of the anti-rat secondary antibody to labeled rat antibodies. Between each step, sections were thoroughly washed multiple times in PBS and rinsed with 0.05% Triton X-100.

Sections were mounted in Dako Fluorescence Mounting Medium. Images were acquired using an LSM900 confocal microscope (20× objective lens, 1024×1024 resolution, averaging: 4, speed: 7, thickness: 1.1-1.3 μm) and processed with ZEN Microscopy software. Quantification was manually performed using 3–5 randomly captured images per section with images. Contact rates were assessed by calculating the percentage of CD4^+^ cells with nuclei that were in contact with the signal of each lineage-specific marker. Cell numbers were obtained by counting marker-positive cells with nuclei. Minimum distances between cells were manually measured using ZEN Microscopy software.

### Isolation of ear skin cells

Ears were split into dorsal and ventral halves, minced, and incubated in 500 μl RPMI1640 medium (Nacalai Tesque, Kyoto, Japan) supplemented with 200 μg/ml Liberase TM (Merck KGaA, Darmstadt, Germany), 1 mg/ml hyaluronidase (Tokyo Chemical Industry), and 200 μg/ml DNAse I (Merck) in microtubes (one ear per tube). Samples were incubated with constant rotation at 37°C for 2.5 hours in an incubator. To detect T_RM_ cytokine production, 10 μg/ml brefeldin A (Merck) was added to the enzyme solution, and the incubation was extended to 3.5 hours. Digested ears were diluted with RPMI1640 medium supplemented with 10% fetal bovine serum, 50 μM 2-mercaptoethanol (FUJIFILM Wako), and 1×penicillin-streptmycin mixed solution (Nacalai Tesque), and were then filtered through 70-μm cell strainers while being mashed using the plungers of 10-ml syringes. Cells were centrifugated, resuspended in 1 ml of 0.1% BSA in PBS, filtered through 30-μm cell strainers, and used in subsequent analyses.

### Flow cytometry

Cells were incubated on ice for more than 20 minutes with 20% rabbit serum and 5 μg/ml anti-CD16/32 antibody (clone; 93) for blocking, followed by an incubation with labeled antibodies as described below.

The following antibodies were used for myeloid cell staining: anti-Ly-6C-FITC (HK1.4, BioLegend), CCR2-PE (475301, R&D systems), CD3ε-biotin (145-2C11, BD Biosciences, San Jose, CA), CD19-biotin (6D5, BioLegend), Ly-6G-biotin (1A8, BioLegend), CD24-PE-Cy7 (M1/69, BioLegend), Tim-4-PE-Cy7 (RMT4-54, BioLegend), Rat IgG2a/κ isotype control-PE-Cy7 (RTK2758, BioLegend), Folr2-APC (10/FR2, BioLegend), Rat IgG2a/κ isotype control-APC (RTK2758, BioLegend), CD45.2-APC-Vio770 (REA737, Miltenyi Biotec), MHC-II-BV421 (M5/114.15.2, BioLegend), CD64-BV605 (X54-5/7.1, BD Biosciences), and CD11b-BV786 (M1/70, BioLegend).

The following antibodies were used for T_RM_ staining: anti-CD103-FITC (2E7, BioLegend), CD4-PE (RM4-4, BioLegend), TCRβ-PerCP-Cy5.5 (H57-597, Tonbo Biosciences, San Diego, CA), CD3ε-PE-Cy7 (145-2C11, BioLegend), CD69-APC (H1.2F3, BioLegend), CD45.2-APC-Vio770, CD8α-biotin (5H10, Thermo Fisher Scientific, Waltham, MA), and CD11b-BV786.

Biotinylated antibodies were detected by further staining with streptavidin-PerCP-Cy5.5 (Thermo Fisher Scientific) or -V500 (BD Biosciences) for myeloid cell staining, and streptavidin-BV605 (BD Biosciences) for T_RM_ staining. As a dilution and wash buffer, 0.1% BSA in PBS was used. In some analyses, cells were finally fixed with 2% PFA.

Regarding T_RM_ cytokine staining, after surface staining, cells were fixed and permeabilized with the Cytofix/Cytoperm Fixation/Permeabilization Kit (BD Biosciences), followed by staining with anti-tumor necrosis factor-α (TNF-α)-FITC (MP6-XT22, BioLegend), rat IgG1/κ isotype control-FITC (HRPN, Tonbo Biosciences), interferon-γ (IFN-γ)-APC (XMG1.2, BioLegend), and rat IgG1/κ isotype control-APC (HRPN, BioLegend).

Concerning unfixed cells, dead cells were stained with propidium iodide (Merck). If cells were fixed, dead cells were stained with Zombie Aqua (BioLegend) before blocking. A flow cytometric analysis was performed using FACSCelesta (BD Biosciences), and data were analyzed with Flowjo software (TreeStar).

### Depletion of colony stimulating factor 1 receptor-dependent myeloid cells

After the resolution of CHS in both ears (>day 35), mice were intraperitoneally injected with 200 μg of an anti-CSF1R antibody (AFS98, Selleck Biotechnology, Kanagawa, Japan) three times a week for a total of 10 doses. As the control, mice were injected with the same amount of normal rat IgG (Rockland Immunochemicals, Limerick, PA) dialyzed in PBS. Two days after the final administration, the right healed ears were rechallenged with 40 mM TNCB. After 3.5 hours, both the untreated left healed ears and the rechallenged right healed ears were processed for enzymatic digestion to isolate ear skin cells.

### Statistical analysis

Each experiment was repeated at least twice with similar results, and representative results are shown unless otherwise noted. Statistical analyses were performed using Prism software (GraphPad) for a one way-ANNOVA with a *post hoc* test or Microsoft Excel for other analyses, with significance defined as *p* < 0.05.

## Results

### Long-term persistence of CD103^+^CD4^+^ T cells in CHS-healed skin

**Figure 1:**
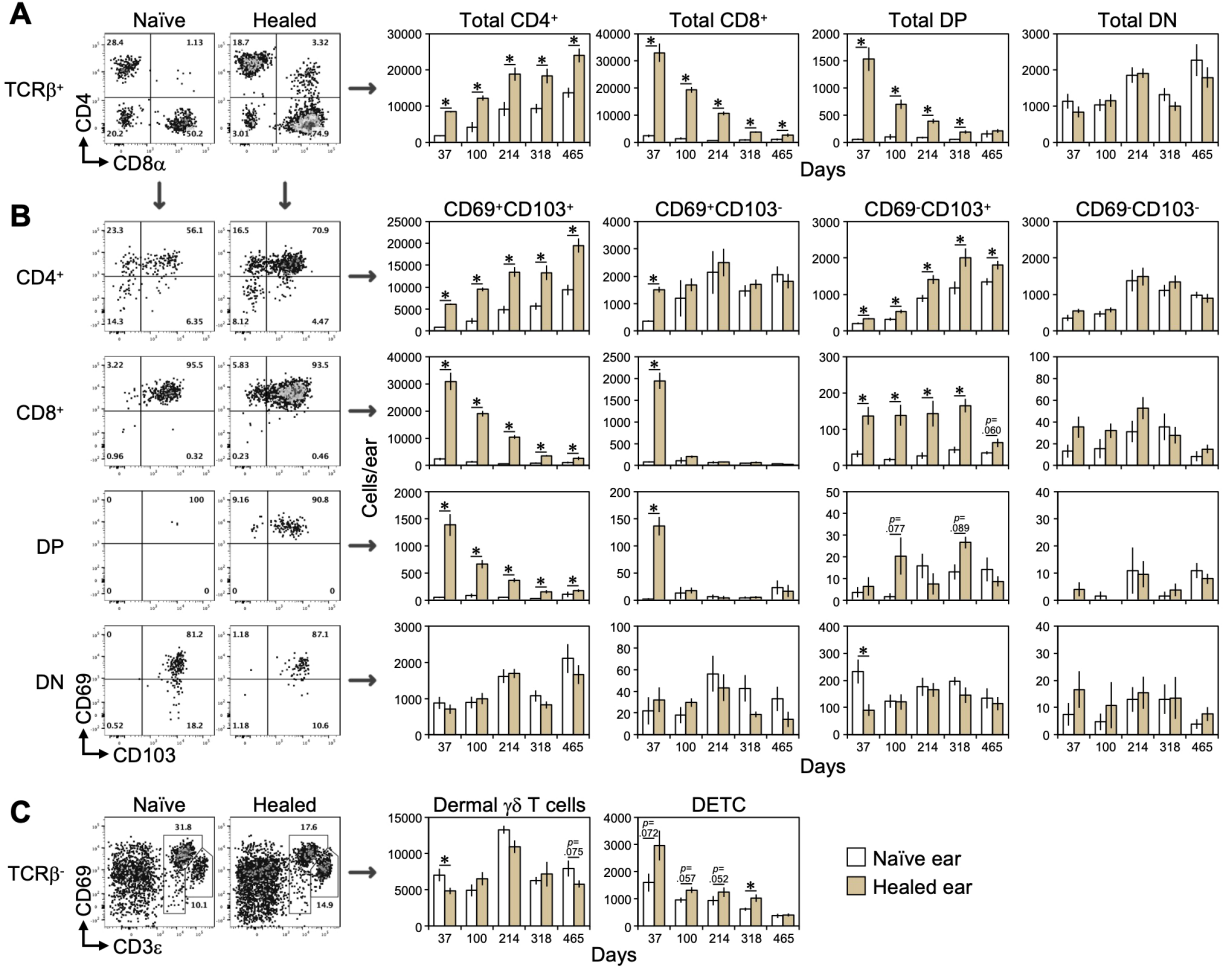
Long-term persistence of CD103^+^CD4^+^ T cells in CHS-healed skin. The right ears of BALB/c mice were sensitized (day -6) and challenged (day 0) with TNCB to induce CHS. Ear swelling peaked on day 1 and resolved by day 35. **(A)** Absolute numbers of CD4^+^, CD8^+^, CD4^+^CD8^+^ DP, and CD4^-^CD8^-^ DN T cells within the αβ T cell fraction (CD45^+^CD11b^-^TCRb^+^) in the naïve (left) and healed (right) ear skin of BALB/c mice. **(B)** Each αβ T cell fraction in **(A)** was further divided by the expression of CD69 and CD103 and the absolute numbers of each fraction are shown. **(C)** The absolute numbers of dermal γδ T cell (CD3ε^lo^) and DETC (CD3ε^hi^) within the γδ T cell fraction (CD45^+^CD11b^-^TCRb^-^). Representative results are shown for experimental results conducted multiple times around days 37 and 100, while data from day 214 onward are from a single experiment. Data represent the mean ± S.E. (n = 5-6, each). *p < 0.05 (naïve left ears vs healed right ears, two-tailed paired *t-*test).

Our previous study using a skin section analysis revealed the long-term persistence of CD4^+^ T cells, but not CD8^+^ T cells, in CHS-healed skin. However, overall changes in skin T cell lineages and their relationship with the expression of the T_RM_ markers CD69 and CD103 remain unclear. To address these issues, we performed a detailed analysis of skin T cell lineages using flow cytometry (**Figure 1**; **Supplementary Figure 1A**). We induced CHS by applying the hapten TNCB to the right ears of BALB/c mice twice, on day -7 or -6 (sensitization) and day 0 (challenge). Ear swelling peaked on day 1 and resolved by day 35 (20).

In the αβ T cell fractions, the number of CD4^+^ and CD8^+^ T cells was higher in freshly healed skin (day 37) than in naïve ear skin (**Figure 1A**). Consistent with our previous skin section analyses, CD4^+^ T cells remained elevated even on day 465, whereas CD8^+^ T cells gradually declined over time. Moreover, CD4^+^ T cells in both naïve and healed skin continued to increase throughout the observation period. CD4^+^CD8^+^ double-positive (DP) T cells, which were absent in naïve skin, emerged in healed skin and declined over time, similar to CD8^+^ T cells. In contrast, CD4^-^CD8^-^ double-negative (DN) T cells remained at similar levels in both naïve and healed skin at all time points.

We then characterized each αβ T cell subset based on CD69 and CD103 expression (**Figure 1B**). In CD4^+^ T cells, CD69^+^CD103^+^ cells predominantly increased and persisted in healed skin, while a small subset of CD69^-^CD103^+^ cells also expanded and was maintained (**Figure 1B**), suggesting that these fractions included CD4^+^ T_RM_ cells, which accounted for approximately 80% of all CD4^+^ T cells in healed skin. The majority of CD8^+^ and DP T cells were CD69^+^CD103^+^ and showed a similar decline over time. In CD4^+^, CD8^+^, and DP T cells, only CD69^+^CD103^-^ cells increased on day 37, suggesting they were the remaining effector T cells that died by day 100.

Skin γδ T cells include CD3^lo^ dermal γδ T cells and CD3^hi^ dendritic epidermal T cells (DETC) (34). Neither population exhibited a significant increase in healed skin.

CD4^+^ T cells in naïve skin are circulating memory T cells despite CD69 and CD103 expression, whereas those in healed skin arise from both increased influx and *bona fide* T_RM_ formation, with the latter being resistant to antibody-mediated depletion (15, 23, 31). The anti-Thy1.2 antibody treatment consistently reduced CD4^+^ T cells in naïve, but not CHS-healed skin, indicating the presence of CD4^+^ T_RM_ cells in CHS-healed skin (**Supplementary Figure 1B**).

These results indicate that only CD4^+^ T_RM_ cells, included in CD103^+^ fractions, persisted in the long term and sustained the enhanced local antigen sensitivity of healed skin.

### Most skin CD4^+^ T cells colocalize with cells expressing macrophage-associated markers

**Figure 2:**
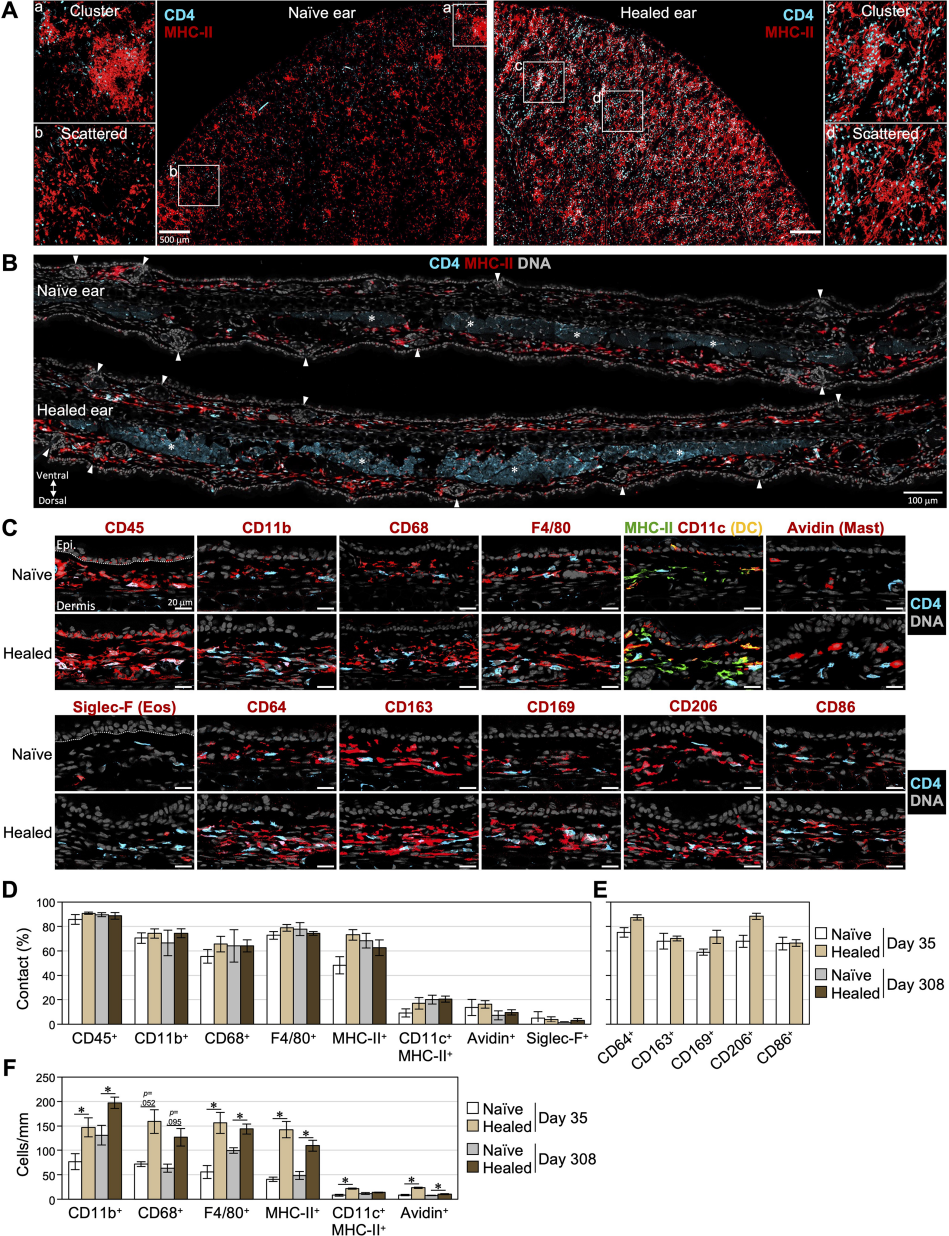
Most skin CD4^+^ T cells are scattered and are in contact with cells expressing macrophage-associated markers. **(A)** Dorsal ear halves of naïve and healed ears on day 35 were stained with CD4 and MHC-II. Z-stack images of large areas (thickness: 10 μm) and within the a-d frame (thickness: 25 μm) were taken from the cartilage side. Bars: 500 μm. **(B)** Naïve and healed ear sections on day 35 were stained with CD4 and MHC-II. DNA was stained with DAPI to visualize nuclei. Arrowheads denote hair follicles and asterisks denote the non-specific background fluorescence of muscle layers. **(A, B)** Representative images from 3–4 mice are shown. **(C-F)** Naïve and healed ear skin sections on days 35 and 308 were stained with the indicated markers. **(C)** Representative images on day 35. Bars: 20 μm. **(D, E)** Percentages of scattered CD4^+^ T cells in contact with cells expressing the indicated markers. **(F)** The numbers of cells expressing the indicated markers in the dermis were counted along the cartilage and were shown as cells/mm. Data represent the mean ± S.E. (n = 3-6, for each). *P < 0.05 (naïve left ears vs healed right ears, two-tailed paired *t-*test).

To reveal cellular niches for the maintenance and reactivation of skin CD4^+^ T_RM_ cells, we examined cells continuously in contact with CD4^+^ T cells in healed skin. Whole mount staining of ear skin on day 35 showed increases in CD4^+^ T cells and MHC-II^+^ APCs in the dermis of healed ears (**Figure 2A**). Some CD4^+^ T cells formed clusters with APCs; however, many were scattered and in contact with APCs. Skin section staining also showed that clusters of CD4^+^ T cells and CD11c^+^MHC-II^+^ APCs were rare on days 35 and 308 (**Figure 2B**, **Supplementary Figures 2A, B**). We evaluated cells that colocalized with scattered CD4^+^ T cells in confocal images of 1.1-to 1.3-μm-thick sections of naïve and healed skin on days 35 and 308 using several lineage markers. Colocalization was defined as visually continuous or touching fluorescent signals in the same focal plane, suggestive of physical contact between cells. On day 35 in healed skin, we found that approximately 90% of CD4^+^ T cells colocalized with CD45^+^ immune cells (**Figures 2C, D**). Approximately 70% of CD4^+^ T cells colocalized with CD11b^+^, CD68^+^, and F4/80^+^ dermal myeloid cells and MHC-II^+^ APCs (**Figures 2C, D**, **Supplementary Figure 2C**). Orthogonal projections of a z-stack image confirmed a close association between CD4^+^ and CD68^+^ cells (**Supplementary Figure 2D**). In contrast, less than 20% of CD4^+^ T cells colocalized with CD11c^+^MHC-II^+^ DCs, Avidin-stained mast cells (35), and Siglec-F^+^ eosinophils (**Figures 2C, D**). There were few CD19^+^ B cells and NKp46^+^ NK cells in the healed skin section (data not shown). Interestingly, CD4^+^ cells in healed skin on day 308 and in naïve skin on days 35 and 308 exhibited a similar pattern of colocalization with each cell lineage.

Since F4/80 was associated with macrophages, we evaluated other macrophage-associated molecules (**Figure 2E**) (36–40). Approximately 70% of CD4^+^ T cells colocalized with cells expressing CD64 (FcγRI), CD163 (scavenger receptor), CD169 (Siglec-1), CD206 (mannose receptor), and CD86 (costimulatory molecule) in naïve and healed skin on day 35.

In addition, many dermal CD4^+^ T cells appeared to colocalize with diverse CD45^-^ stromal cells, including nerve bundles, blood vessels, muscle, and dermal fibroblasts (**Supplementary Figure 3**).

These results indicate the long-term maintenance of skin CD4^+^ T_RM_ cells in contact with dermal myeloid cells, including macrophages.

### Transient expansion of dermal myeloid cells and sustained increase in cDC1 in healed skin

**Figure 3:**
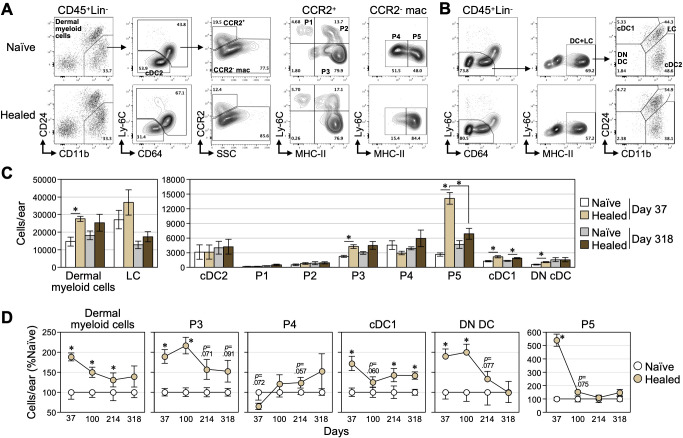
Temporal changes in the number of skin myeloid cells after the resolution of CHS. **(A, B)** Representative flow cytometry plots of isolated naïve and healed ear skin cells on day 35 post-challenge. **(A)** CD11b^+^ dermal myeloid cells were analyzed according to previous studies (41–43). CD11b^+^CD24^lo/-^ dermal myeloid cells in CD45^+^Lineage^-^ (Lin^-^: CD3ε^-^CD19^-^ Ly-6G^-^) cells contained CD64^-^Ly-6C^-^ cells (cDC2) and remaining CCR2^+^ and CCR2^-^ populations. Based on Ly-6C and MHC-II expression, CCR2^+^ cells were divided into dermal monocytes (P1) and Ly-6C^+^ (P2) and Ly-6C^-^ (P3) MoDCs/CCR2^+^ macrophages. CCR2^-^ cells consisted of MHC-II^lo/-^ (P4) and MHC-II^hi^ (P5) macrophages. **(B)** DCs and LC populations were analyzed according to previous studies (41, 44). CD45^+^Lin^-^CD64^-^Ly-6C^-^MHC-II^hi^ cells consisted of CD24^+^CD11b^lo/-^ (cDC1), CD24^+^CD11b^+^ (LCs), CD24^-^CD11b^+^ (cDC2), and CD24^-^CD11b^-^ (DN cDCs). **(C, D)** The numbers of each cell lineage in naïve and healed ear skin were analyzed. **(C)** Absolute cell numbers were compared between days 37 and 318. **(D)** Temporal changes in the relative numbers of each myeloid cell population (expressed as a percentage relative to those in naïve skin) were analyzed at the indicated time points. Representative results are shown for experimental results conducted multiple times around days 37 and 100, while data from day 214 onward are from a single experiment. Data represent the mean ± S.E. (n = 5-6, each). *P < 0.05 [two-tailed paired (naïve vs healed) and unpaired *t-*test (days 37 vs 318)].

Skin section staining on days 35 and 308 revealed a sustained increase in dermal myeloid cells expressing CD11b, CD68, and F4/80, along with slight and transient increases in DCs and mast cells in CHS-healed ear skin (**Figure 2F**); however, the changes in their composition remain unclear. According to the methods of fractionation of CD11b^+^ dermal myeloid cells (41–43) and DCs (41, 44), we investigated which myeloid cell populations were affected by CHS. CD11b^+^CD24^-^ dermal myeloid cells contained cDC2s (CD64^-^Ly-6C^-^), P1 monocytes (CCR2^+^Ly-6C^+^MHC-II^-^), P2 (CCR2^+^Ly-6C^+^MHC-II^+^) and P3 (CCR2^+^Ly-6C^-^MHC-II^+^) monocyte-derived DCs (MoDCs), and P4 MHC-II^lo^ (CCR2^-^Ly-6C^-^) and P5 MHC-II^hi^ (CCR2^-^Ly-6C^-^) macrophages (**Figure 3A**). The classification of MoDCs is based on their dependency on Flt3 signaling (43), whereas other studies refer to the P2+P3 population as CCR2^+^ macrophages based on CD64 expression (45). Therefore, we herein referred to the P2 and P3 populations as MoDCs/CCR2^+^ macrophages. In addition, the CD64^-^MHC-II^+^ population contained type I conventional DCs (cDC1, CD11b^-^CD24^+^), cDC2s (CD11b^+^CD24^-^), and DN cDCs (CD11b^-^CD24^-^), in addition to LCs (CD11b^+^CD24^+^), an epidermal macrophage population (**Figure 3B**) (46, 47).

**Figure 4:**
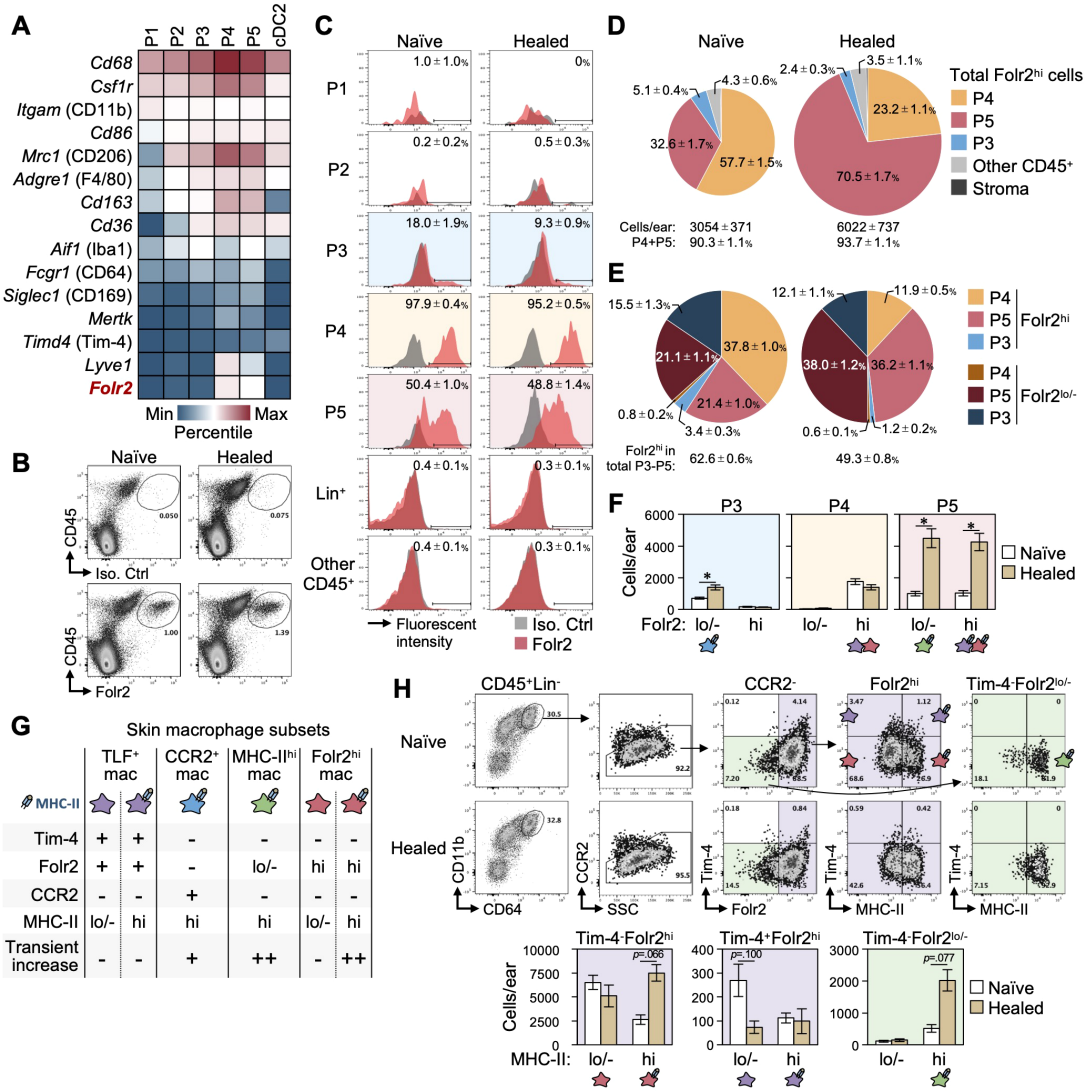
Folr2^hi^ macrophages are the major macrophage population in skin. **(A)** The expression of common macrophage marker genes in dermal myeloid cell lineages in mouse naïve ear skin was obtained from a public database (43). The heat map shows microarray expression values (color scale based on percentiles). **(B-F)** The frequency of Folr2^hi^ cells in naïve and healed ear skin cells on day 38 was analyzed. Representative plots of CD45 and Folr2 expression in whole ear skin cells **(B)**, and Folr2 expression in P1-P5, Lin^+^ (CD3ε^+^CD19^+^Ly-6G^+^), and other remaining CD45^+^ cell fractions **(C)**. **(D)** Pie charts show the distribution of total Folr2^hi^ cells to the indicated fractions. The size of pie charts represents the number of cells. “Other CD45^+^” and “stroma” fractions represent CD45^+^ cells other than the P3-P5 fractions and CD45^-^ cells, respectively. **(E)** Distribution of Folr2^hi^ and Folr2^lo/-^ cells in total P3-P5 fractions. **(F)** Numbers of Folr2^hi^ and Folr2^lo/-^ cells in P3-P5 fractions. Data are the mean ± S.E. (n = 5, for each). *P < 0.05 (naïve left ears vs healed right ears, two-tailed paired *t-*test). **(G)** Classification of skin macrophage populations proposed by Dick et al. (45) and found in the present study. **(H)** Tim-4, Folr2, and MHC-II expression in the CD45^+^Lin^-^CD11b^+^CD64^+^CCR2^-^ macrophage population in naïve and healed ear skin on day 50. Graphs represent the numbers of MHC-II^hi^ and MHC-I^lo/-^ populations in the indicated fractions (mean ± S.E., n = 5, for each). *P* values (naïve left ears vs healed right ears, two-tailed paired *t-*test). **(F, H)** The macrophage subset included in each population is shown at the bottom of the graphs.

We examined the absolute numbers of (**Figure 3C**) and temporal changes in the relative numbers (**Figure 3D**) of myeloid cell populations. On day 37, a significant increase was observed in dermal myeloid cells in freshly healed skin (**Figure 3C**). P5 MHC-II^hi^ macrophages exhibited the most significant increase, while P3 MoDCs/CCR2^+^ macrophages, cDC1s, and DN DCs showed slight increases. The numbers of LCs, cDC2s, P1 monocytes, P2 MoDCs/CCR2^+^ macrophages, and P4 MHC-II^lo^ macrophages did not significantly change. The numbers of P5 MHC-II^hi^ macrophages, P3 MoDCs/CCR2^+^ macrophages, and DN cDCs decreased to a similar level as those in naïve skin after days 100 and 214, respectively (**Figures 4C, D**). cDC1s still showed a slight increase on day 318.

These results indicate that CHS increased certain populations of myeloid cells and DCs in the dermis (primarily P5 MHC-II^hi^ macrophages) and these changes (except for cDC1) were transiently maintained for a period following healing.

### Folr2^hi^ macrophages are the major macrophage population in skin

An analysis of macrophage-associated gene expression in isolated dermal myeloid cell populations in naïve skin from a public database (43) showed that many of the genes were expressed not only in P4 and P5 macrophage fractions, but also in the P1-P3 and/or cDC2 fractions (**Figure 4A**, *CD68*-*Mertk*). Therefore, it remains unclear whether CD4^+^ T_RM_ cells are truly in contact with macrophages in skin. We searched for genes specifically expressed in P4 and P5 macrophages and identified *Folr2* (Folate receptor β) (**Figure 4A**). A flow cytometric analysis of isolated skin cells revealed that Folr2^+^ cells were exclusively CD45^+^ immune cells in both naïve and healed skin (**Figure 4B**). Among dermal myeloid cells, Folr2^+^ cells were primarily found in P4 and P5 macrophage fractions, with a smaller subset (10-20%) in the P3 MoDCs/CCR2^+^ macrophage fraction (**Figure 4C**). Almost all P4 MHC-II^lo^ macrophages were Folr2^hi^, whereas 50% of P5 MHC-II^hi^ macrophages were Folr2^hi^ and the remaining 50% were Folr2^lo/-^ (**Figure 4C**). Only background levels of Folr2^+^ cells were detected in other immune and stromal cell fractions (**Figures 4B–D**). Analyses of the total numbers of Folr2^hi^ cells revealed that more than 90% of these cells were contained within P4+P5 fractions in both naïve and healed skin (**Figure 4D**). In addition, Folr2^hi^ cells represented 62.6 and 49.3% of the total P3-P5 populations in naïve and healed skin, respectively (**Figure 4E**). On day 38, Folr2^hi^ and Folr2^lo/-^ cells in the P5 MHC-II^hi^ macrophage fraction both increased in healed skin (**Figure 4F**). Folr2^lo/-^, but not Folr2^hi^ cells in the P3 MoDCs/CCR2^+^ macrophage fraction increased in healed skin (**Figure 4F**). In contrast, no changes were observed in Folr2^lo/-^ or Folr2^hi^ cells in the P4 MHC-II^lo^ macrophage fraction. These results demonstrate that Folr2 serves as a highly specific marker for P4 MHC-II^lo^ and 50% of P5 MHC-II^hi^ macrophages among cells in skin.

CD11b^+^CD64^+^ tissue macrophages across organs were recently classified into three subsets based on their gene expression and life cycle properties; TLF^+^ macrophages (Tim-4^+^Lyve-1^+^Folr2^+^) that emerge during the embryonic period and are maintained by self-renewal, CCR2^+^ macrophages (Tim-4^-^Lyve-1^-^Folr2^-^) that are constantly replaced by monocytes and correspond to the P2 and P3 fractions in the present study, and MHC-II^hi^ macrophages (Tim-4^-^Lyve-1^-^Folr2^-^CCR2^-^) that show intermediate replacement by monocytes (45). Skin macrophages may also be divided into these 3 populations with the surface staining of Tim-4 and CCR2 (**Figure 4G**); however, the relationship between Folr2^hi^ macrophages in P4 and P5 fractions and TLF^+^ macrophages remains unclear.

Therefore, we herein examined the expression of Folr2, Tim-4, and MHC-II in the CCR2^-^ macrophage population on day 50 post-challenge (**Figure 4H**). We found that the Folr2^hi^ population contained only a small fraction of TLF^+^ macrophages (Tim-4^+^Folr2^hi^ cells). As a positive control for Tim‑4 staining, Tim‑4⁺ cells were detected in a subset of DCs (**Supplementary Figure 4**), consistent with a previous report (48). The further fractionation of the Folr2^hi^ population revealed that the Tim-4^+^ and Tim-4^-^ fractions may both be subdivided into MHC-II^hi^ and MHC-II^lo^ subsets. In the Tim-4^+^ fraction, the number of MHC-II^hi^ cells remained unchanged, whereas that of MHC-II^lo/-^ cells slightly decreased in healed skin. In the Tim-4^-^ fraction, similar to the P4/P5 Folr2^hi^ populations shown in **Figure 4F**, only the MHC-II^hi^ subset increased. MHC-II^hi^ cells in the Folr2^lo/-^ fraction corresponded to MHC-II^hi^ macrophages described by Dick et al. (45) and P5 Folr2^lo/-^ macrophages in **Figure 4F**.

These results indicate additional heterogeneity within skin macrophages and suggest that Tim-4^-^Folr2^hi^ macrophages constitute the major macrophage population in both naïve and healed skin. To distinguish Tim-4^-^Folr2^hi^ macrophages from TLF^+^ macrophages, we herein referred to them as Folr2^hi^ macrophages (**Figure 4G**).

### Skin CD4^+^ cells are maintained in contact with Folr2^hi^ macrophages

**Figure 5:**
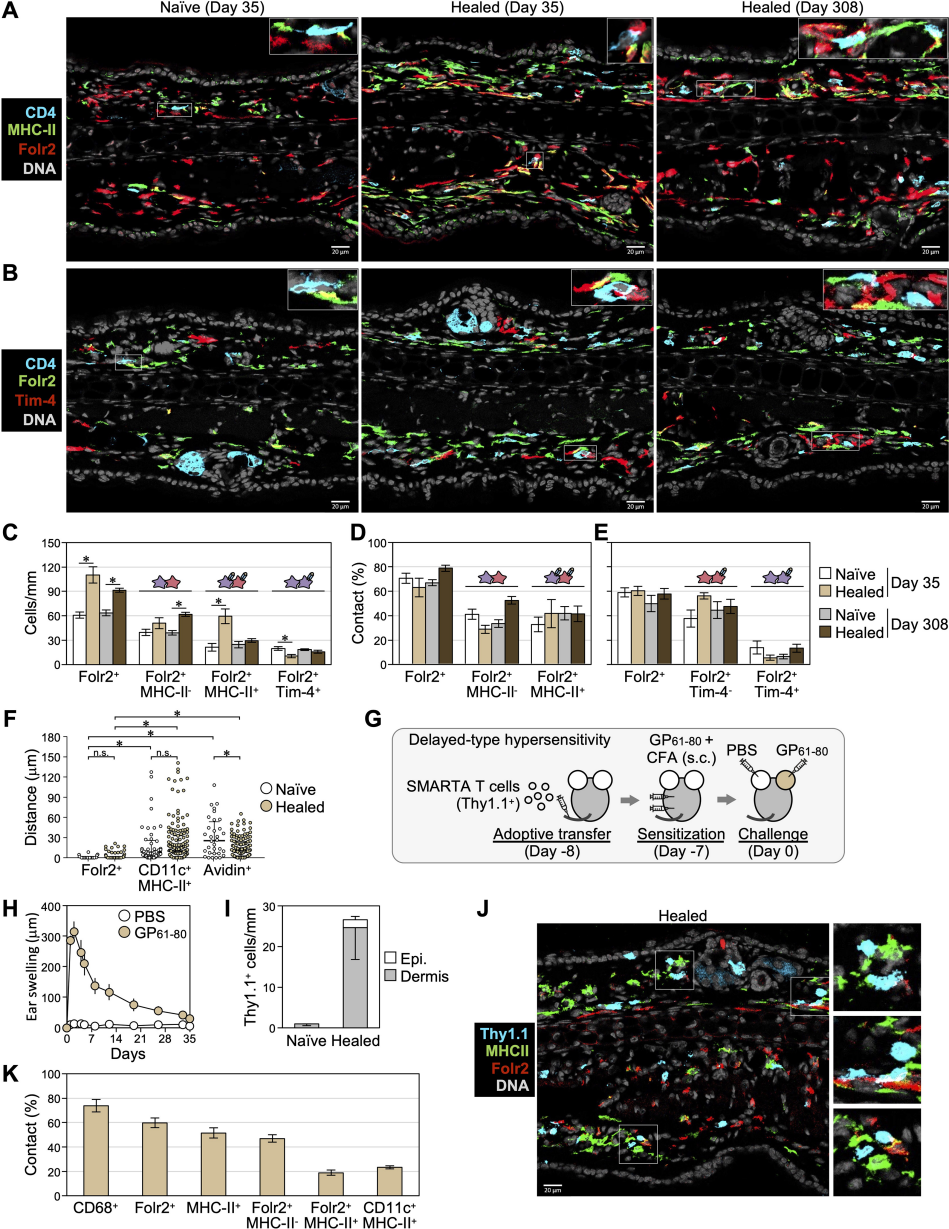
Skin CD4^+^ T cells are maintained in contact with Folr2^hi^ macrophages. **(A, B)** Representative images of naïve and healed ear skin sections on days 35 and 308 stained with the indicated markers. **(C)** The numbers of cells expressing the indicated markers in the dermis were counted along the cartilage. *P < 0.05 (naïve vs healed, two-tailed paired *t-*test). **(D, E)** Percentages of scattered CD4^+^ T cells in contact with cells expressing the indicated markers. **(C-E)** Data represent the mean ± S.E. (n = 6 for day 35, n = 5 for day 308). **(F)** The minimum distances between individual dermal CD4^+^ T cells and the nearest Folr2^+^ macrophages, CD11c^+^MHC-II^+^ DCs, or avidin^+^ mast cells in sections on day 35 were measured (n = 31–45 for naïve skin, n = 132–148 for healed skin, data combined from 3 mice per group). Bars: median ± interquartile range. *P < 0.05 (a one-way ANOVA with Bonferroni *post-hoc* comparisons tests). **(G-K)** DTH mediated by transferred LCMV GP_61-80_-specific SMARTA CD4^+^ T cells (Thy1.1^+^) was induced as depicted in **(G)**. **(H)** Ear swelling after the challenge by day 35. **(I-K)** Naïve and healed ear skin sections on day 35 were stained with the indicated molecules in **(J)**. **(I)** The numbers of epidermal and dermal SMARTA CD4^+^ T cells in naïve and healed ear skin were counted along the cartilage. **(K)** Percentages of SMARTA CD4^+^ T cell in contact with cells the expressing indicated markers. The combined data of two experiments are shown as mean ± S.E. (n = 6).

The colocalization of CD4^+^ T cells with Folr2^+^ and TLF^+^ macrophages was analyzed (**Figures 5A–F**). Consistent with flow cytometry analyses shown in **Figures 3**, **4F**, Folr2^+^MHC-II^-^ cells (corresponding to P4 Folr2^hi^MHC-II^lo/-^ macrophages) and Folr2^+^MHC-II^+^ cells (corresponding to P5 Folr2^hi^MHC-II^hi^ macrophages and a small subset of P3 Folr2^hi^MHC-II^hi^ cells) were observed in sections, and Folr2^+^MHC-II^+^ cells increased in healed skin on day 35 and decreased by day 308 (**Figures 5A, C**). Furthermore, similar to the contact rates of CD4^+^ T cells to cells expressing macrophage-related molecules shown in **Figure 2**, approximately 70% of CD4^+^ T cells colocalized with Folr2^+^ cells in both naïve and healed skin on days 35 and 308 (**Figure 5D**). Approximately 40% of CD4^+^ T cells colocalized with Folr2^+^MHC-II^+^ macrophages, while another 40% were in contact with Folr2^+^MHC-II^-^ macrophages (**Figure 5D**). In contrast, as shown in the analysis in **Figure 4H**, Tim-4^+^Folr2^+^ cells (TLF^+^ macrophages) were present in small numbers in the skin (**Figures 5B, C**), and only a minor fraction of CD4^+^ T cells colocalized with Tim-4^+^Folr2^+^ cells (**Figures 5B, E**). We measured the distances between individual CD4^+^ T cells and the nearest myeloid cells in skin sections on day 35. In naïve and healed skin, dermal CD4^+^ T cells associated more closely with Folr2^+^ macrophages than with DCs or mast cells (**Figure 5F**). These results suggest that most CD4^+^ T_RM_ cells were primarily maintained through contact with either MHC-II^hi^ or MHC-II^lo/-^ Folr2^+^ macrophages. In addition, clusters of CD4^+^ T cells and APCs contained only a few Folr2^+^ cells (**Supplementary Figure 2B**), suggesting that DCs and macrophages other than Folr2^+^ macrophages contribute to the formation of rare clusters.

In addition to antigen-specific T cells, CD4^+^ T cells in CHS-experienced skin include circulating endogenous memory T cells (15, 31) and approximately 15% Foxp3^+^ regulatory T cells (20). To assess the interaction between antigen-specific CD4^+^ T cells and macrophages and examine strain- and model-specific differences, we established a DTH model, a T cell-dependent allergic reaction induced by a protein antigen, to evaluate memory T cells derived from transferred antigen-specific CD4^+^ T cells in the healed skin of C57BL/6 mice (**Figure 5G**). Naïve SMARTA CD4^+^ T cells, which express a T cell receptor transgene with specificity for the immunodominant I-A^b^-restricted LCMV GP_61-80_ (33), were transferred into WT C57BL/6 mice. Recipient mice were subcutaneously sensitized with LCMV GP_61–80_ plus CFA, followed by an intradermal injection of LCMV GP_61–80_ into the ear skin, which induced the DTH response that peaked on day 2 (**Figure 5H**). Consistent with observations in the BALB/c mouse CHS model, SMARTA CD4^+^ T cells persisted predominantly in the dermis of freshly healed skin on day 35 (**Figures 5I, J**). Approximately 70% of SMARTA CD4^+^ T cells colocalized with CD68^+^ dermal myeloid cells, 60% Folr2^+^ macrophages, 45% Folr2^+^MHC-II^+^ macrophages, 20% Folr2^+^MHC-II^-^ macrophages, and 20% CD11c^+^MHC-II^+^ DCs (**Figures 5J, K**). These results indicate that although interactions with Folr2^+^MHC-II^+^ macrophages were less frequent, antigen-specific CD4^+^ T cells persisting in C57BL/6 DTH models also primarily interacted with Folr2^+^ macrophages.

### The maintenance and reactivation of skin CD4^+^ T_RM_ cells are not impaired in the absence of CSF1R-dependent myeloid cells, including Folr2^hi^ macrophages

**Figure 6:**
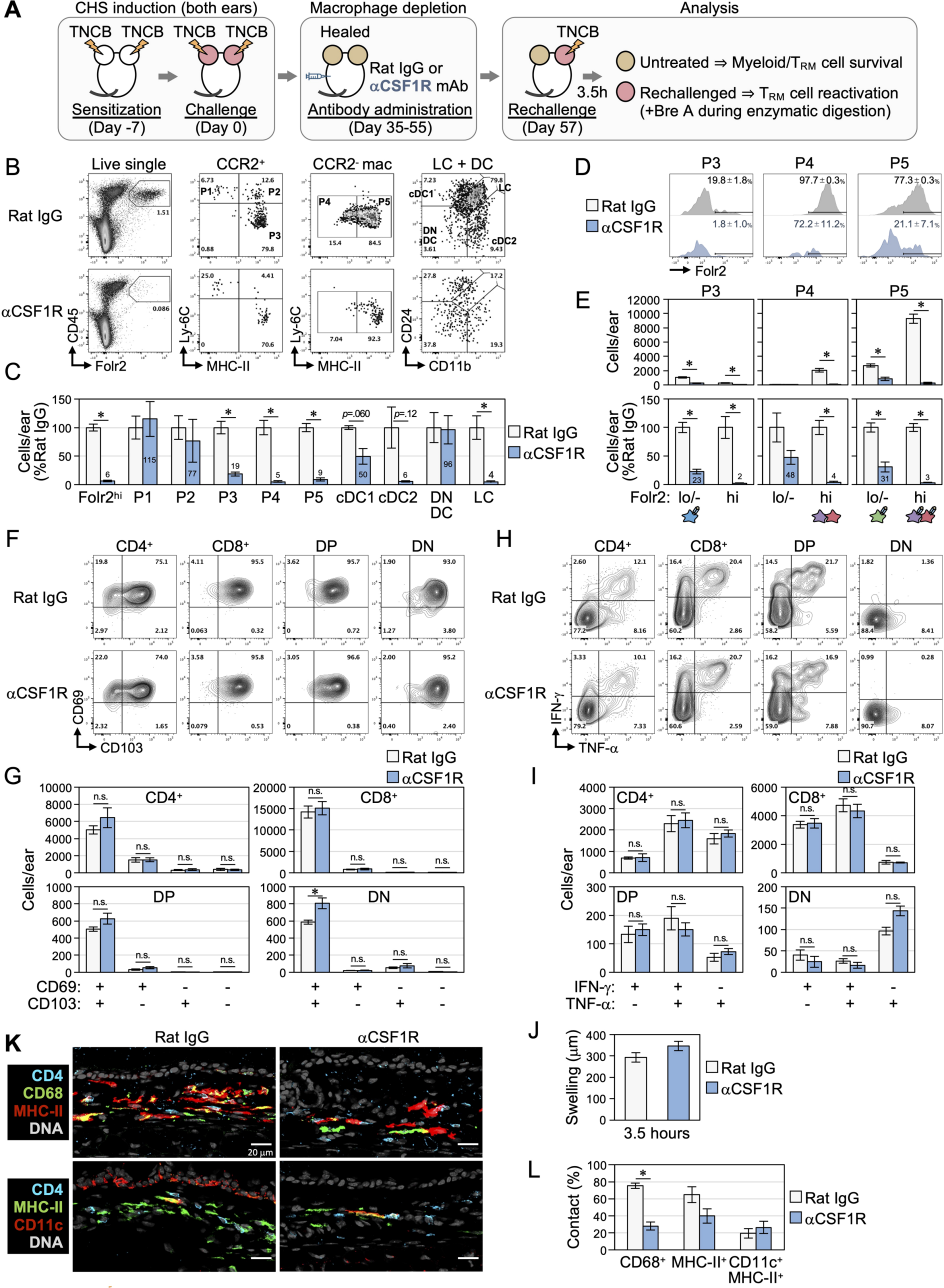
The maintenance and reactivation of skin CD4^+^ T_RM_ cells are intact in the absence of Folr2^hi^ macrophages. **(A-L)** After the resolution of CHS in both ears, BALB/c mice were injected i.p. with an anti-CSF1R antibody (clone: AFS98) or normal rat IgG (for control) 3 times a week for a total of 10 times to deplete CSF1R-dependent myeloid cells. The right ears were then rechallenged with TNCB and untreated left and rechallenged right healed skin was analyzed as depicted in **(A)**. **(B-G)** Myeloid and T_RM_ populations in untreated left healed skin were analyzed. **(B)** Representative flow cytometry plots fractionated as in **Figures 3A, B** and **Figure 4B**. **(C)** The numbers of the indicated populations compared to each control treatment (rat IgG) are shown as the mean ± S.E. (n = 3, each). **(D)** Folr2 expression in P3-P5 fractions, as in **Figure 4C**. Numbers represent the mean ± S.E. (n = 3) of the Folr2^hi^ fraction in each population. **(E)** The absolute (upper) and relative (lower) numbers of Folr2^hi^ and Folr2^lo/-^ cells in P3-P5 populations. The macrophage subset, as in **Figure 4G**, included in each population is shown. **(F, G)** Representative plots and the numbers of each fraction of skin ab T cells divided by CD69 and CD103 expression are shown as the mean ± S.E. (n = 3, each). **(H, I)** Representative plots and the numbers of IFN-γ and TNF-α expression in ab T cell populations in rechallenged right healed ears are shown as the mean ± S.E. (n = 6, each). **(J)** The swelling of right healed ear skin 3.5 hours post-rechallenge is shown as the mean ± S.E. (n = 3, each). **(K, L)** Untreated left healed ear sections were stained with the indicated markers. The percentages of remaining CD4^+^ T cells in contact with cells expressing the indicated markers are shown as the mean ± S.E. (n = 3, each).

To investigate the involvement of Folr2^hi^ macrophages in the survival and reactivation of CD4^+^ T_RM_ as well as CD8^+^ T_RM_ and DP/DN T cells, mice that had experienced CHS on both ears were administered the anti-CSF1R antibody to deplete CSF1R-dependent macrophages (**Figure 6A**) (49, 50). TNCB was then rechallenged on the right ear, and skin cells were enzymatically isolated from both ears 3.5 hours later for analyses. Untreated left healed ears were used to examine the depletion of myeloid cells and the survival of T_RM_ cells, while rechallenged right healed ears were analyzed for T_RM_ cell reactivation.

The administration of the anti-CSF1R antibody successfully depleted 94% of Folr2^hi^ macrophages (**Figures 6B, C**). More than 90% of the P4 (MHC-II^lo/-^ TLF^+^ and Folr2^hi^ macrophages) and P5 (MHC-II^hi^ macrophages, MHC-II^hi^ TLF^+^ and Folr2^hi^ macrophages) fractions were removed. Additionally, most P3 MoDC/CCR2^+^ macrophages, cDC2, and LCs were eliminated, while the number of cDC1s slightly decreased (**Figures 6B, C**). CSF1R dependency was previously reported not only in macrophages, but also in cDC2s and LCs (51–53). Furthermore, comparisons of the Folr2^hi^ and Folr2^lo/-^ populations within P3-P5 fractions revealed that the anti-CSF1R antibody preferentially depleted Folr2^hi^ fractions across all cell populations (**Figures 6D, E**). These results suggest that TLF^+^ and Folr2^hi^ macrophages are more dependent on CSF1R than other macrophage populations.

However, the depletion of CSF1R-dependent myeloid cells did not affect any fractions of CD4^+^ T cells in healed skin, defined by CD69 and CD103 expression (**Figures 6F, G**). This result suggests that although the majority of skin CD4^+^ T_RM_ cells remained in contact with Folr2^hi^ macrophages, all CSF1R-dependent myeloid cells, including Folr2^hi^ macrophages, were unnecessary for the persistence of skin CD4^+^ T_RM_ cells. Similarly, CSF1R-dependent myeloid cells were also dispensable for the maintenance of CD8^+^, DP, and DN T cells.

It currently remains unclear whether the interaction with Folr2^+^ macrophages plays a role in the reactivation of skin CD4^+^ T_RM_ cells. Therefore, we examined T_RM_ cell reactivation by the production of IFN-γ and/or TNF-α upon the rechallenge (20). In rechallenged healed ears, IFN-γ^+^TNF-α^+^ polyfunctional T cells and IFN-γ^+^ or TNF-α^+^ monofunctional T cells were detected in the CD4^+^, CD8^+^, and DP T cell subsets (**Figure 6H**). The percentages of polyfunctional and monofunctional cells were similar between CD8^+^ and DP T cells. A small fraction of DN T cells predominantly exhibited TNF-α^+^IFN-γ^-^ monofunctional activity. Notably, cytokine production by all αβ T cells and ear swelling remained unaffected by the depletion of CSF1R-dependent myeloid cells (**Figures 6H–J**). These results indicate that CSF1R-dependent skin myeloid cell populations, including Folr2^+^ macrophages, are not essential for the reactivation of CD4^+^ T_RM_ cells, CD8^+^ T_RM_ cells, and DN/DP T cells.

We then investigated whether skin CD4^+^ T cells, after the depletion of CSF1R-dependent myeloid cells, established new interactions with the remaining myeloid cells or DCs to maintain their survival and enable reactivation upon antigen re-exposure. The anti-CSF1R antibody treatment markedly reduced the contact frequency between CD4^+^ T cells and CD68^+^ cells, including CD11b^+^ dermal myeloid cell populations (**Figures 6K, L**). The contact frequency between CD4^+^ T cells and the remaining MHC-II^+^ APCs slightly decreased, while no significant change was observed in the contact frequency with CD11c^+^MHC-II^+^ DCs. These results indicate that following the depletion of CSF1R-dependent myeloid cells, CD4^+^ T_RM_ cells previously interacting with Folr2^+^ macrophages did not establish new contacts with the remaining myeloid cells and DCs.

## Discussion

The aim of the present study was to identify the niche cells involved in the maintenance and reactivation of CD4^+^ T_RM_ cells in CHS-healed skin. Based on the hypothesis that cells persistently in contact with scattered CD4^+^ T_RM_ cells in the dermis function as their niche, we found that the majority of CD4^+^ T cells remained in contact with Folr2^hi^ macrophages, while approximately 20% were in contact with DCs in both naïve and healed skin. However, the depletion of skin Folr2^hi^ macrophages and several other myeloid cell populations by the anti-CSF1R antibody revealed that these CSF1R-dependent myeloid cells were not required for the maintenance and reactivation of not only CD4^+^ T_RM_ cells, but also all other skin αβ T_RM_ cell populations.

The present study suggests that long-term local immune memory in CHS-healed skin is mediated by a subset of CD103^+^CD4^+^ T_RM_ cells that persist over time. Furthermore, these cells continue to increase over time after healing. We previously demonstrated that the antigen responsiveness of both naïve and healed skin upon an antigen rechallenge increased over time (20), suggesting that the influx of antigen-specific circulating memory CD4^+^ T cells and their differentiation into T_RM_ cells, as well as the local proliferation of pre-existing CD4^+^ T_RM_ cells, were progressive. However, other studies that examined temporal changes in mouse skin CD4^+^ T_RM_ cell numbers did not report this increase (31, 32). Although the number of CD4^+^ T cells has been shown to increase with aging in human skin (54–56), the relationship between these findings and the present results remains unclear.

We also found that CD4^+^CD8^+^ DP T cells increased in CHS-healed skin. DP T cells have been reported to originate from either activated CD4^+^ and CD8^+^ T cells under chronic inflammatory conditions, where they may contribute to disease pathogenesis (57–59). Although the origin of DP T cells in CHS-healed skin remains unclear, they exhibited a phenotype resembling CD8^+^ T_RM_ cells, characterized by CD69 and CD103 expression, gradually declined over time, and produced IFN-γ and TNF-α upon reactivation. These results indicate that DP T_RM_ cells, which share properties with CD8^+^ T_RM_ cells, also form in transient inflammation, such as CHS. Additionally, the numbers of CD4^-^CD8^-^ DN T cells remained unchanged regardless of CHS experience or time progression. DN T cells have been reported to exhibit immunosuppressive functions (60–62); however, their role in the regulation of CHS remains unclear. Collectively, the present results indicate that the enhanced antigen responsiveness of CHS-experienced skin is initially mediated by CD4^+^, CD8^+^, and DP T_RM_ cells immediately after healing, but progressively shifts over time to a response predominantly driven by CD4^+^ T_RM_ cells.

Collins et al. demonstrated that following the resolution of inflammation in trunk skin, CCR2-dependent monocyte-derived DCs and macrophages remained elevated for at least 130 days (14). In CHS-experienced ear skin, based on the classification of dermal myeloid cell subsets (41–43) and Folr2 expression, we identified the expanding populations as Folr2^hi^ and Folr2^lo^ macrophages (P5 MHC-II^hi^ macrophage fraction), P3 MoDCs/CCR2^+^ macrophages, and cDC1 and CD11b^-^CD24^-^ DN cDCs. Dick et al. proposed a classification of tissue macrophages across organs based on Tim-4 and CCR2 surface expression, linking these markers to differences in gene expression and/or cellular life cycles. Their classification included TLF^+^ macrophages (Tim-4^+^CCR2*^-^*), CCR2^+^ macrophages (Tim-4^-^CCR2^+^), and MHC-II^+^ macrophages (Tim-4^-^CCR2*^-^*) (45). However, in the context of skin macrophages, their study focused solely on the classification of these fractions with Tim-4 and CCR2 surface expression and their life cycles, leaving other gene expression profiles, including Folr2, unexamined. We found that skin macrophages contained only a small fraction of Tim-4^+^Folr2^hi^ macrophages, corresponding to TLF^+^ macrophages, while the majority belonged to the Tim-4^-^Folr2^hi^ macrophage subset (including the MHC-II^hi^ and MHC-II^lo/-^ populations), which falls under the MHC-II^hi^ macrophage fraction (Tim-4^-^CCR2*^-^*) according to the classification reported by Dick et al. (45). These findings suggest that the Folr2^hi^ macrophage subset, as defined in the present study, represents the largest macrophage population in the skin. Although the mechanisms underlying the increases in Folr2^hi^ macrophages and other myeloid cell populations in CHS-experienced skin remain unclear, the CCR2-dependent ongoing recruitment of monocytes and/or local proliferation, as reported by Collins et al., appears to play a role (14).

Skin CD4^+^ T cells have complex traits that complicate their identification. In naïve skin, all CD4^+^ T cells are circulating memory cells, despite expressing CD69 and CD103 (31). In inflammation-resolved skin, their increase results from the enhanced influx of circulating memory T cells and *bona fide* T_RM_ formation (15, 31). Accordingly, the depletion of circulating CD4^+^ T cells reduced their numbers in naïve, but not CHS-healed skin. Similar contact rates between dermal CD4^+^ T cells and myeloid cells in naïve and healed skin suggest that CD4^+^ T_RM_ cells retain their ability to interact with myeloid cells post-differentiation. Further investigations on adhesion molecule and chemokine receptor heterogeneity among CD4^+^ T cell subsets and T_RM_ populations are needed to elucidate the underlying mechanisms.

Perifollicular APC clusters, which may contribute to the maintenance and reactivation of CD4^+^ T_RM_ cells, have been observed in the trunk skin of mice following inflammation (31, 32), as well as in both healthy and inflamed human skin (31, 63, 64). However, other studies have reported a scattered distribution of CD4^+^ T_RM_ cells in the mouse and human trunk skin (18, 54, 55, 65, 66). Consistent with our observations, most dermal CD4^+^ T_RM_ cells in healed ear skin formed by fungal infection have been shown to persist in a scattered pattern while maintaining contact with APCs (15). Additionally, the maintenance of CD4^+^ T_RM_ cells across various organs has been reported to involve clustering with APCs in some cases, while a scattered distribution has been detected in others (30, 67–69). Moreover, skin CD4^+^ T_RM_ cells exhibit high motility in trunk skin, but become sessile in ear skin (15, 31). These findings suggest that the mode of CD4^+^ T_RM_ cell maintenance, including clustering versus dispersion and the presence or absence of migration, varies across tissues and may also differ within the same tissue depending on the context.

In ear skin, the majority of scattered CD4^+^ T cells remained in contact with Folr2^hi^ macrophages; however, these macrophages were not required for their maintenance. Furthermore, following the depletion of CSF1R-dependent myeloid cells, skin CD4^+^ T cells did not establish new interactions with other APCs. These results suggest that single niche cells sustaining the long-term persistence of CD4^+^ T_RM_ cells through stable contact may not exist in CHS-healed ear skin. It is also possible that skin CD4^+^ T_RM_ cells, similar to T_RM_ cell populations in other organs (70, 71), do not require TCR signaling for their maintenance. Therefore, the following possibilities need to be considered: 1) different CD4^+^ T_RM_ cell subsets may rely on distinct cellular niches [i.e., Th17 T_RM_ cells on CD301b^+^ myeloid cells (32)], 2) migratory CD4^+^ T_RM_ cells may be maintained through intermittent interactions with survival factor-producing cells, and 3) sessile CD4^+^ T_RM_ cells may rely on widely expressed or soluble survival factors in the skin.

Skin T_RM_ cell reactivation was also independent of CSF1R-dependent myeloid cells. Tamoutounour et al. showed that P4 and P5 macrophages, including Folr2^hi^ macrophages, were unable to activate antigen-specific CD4^+^ T cells with protein antigens *in vitro* despite their high phagocytic activity, indicating their limited capacity for antigen processing and presentation on MHC-II (43). However, since haptens directly bind to self-peptides on MHC-II, bypassing antigen processing (72), these cells are theoretically capable of antigen presentation in CHS. Nevertheless, MHC-II^hi^Folr2^hi^ macrophages did not contribute to CD4^+^ T_RM_ cell reactivation, and the reason for this remains unclear. After the anti-CSF1R antibody treatment, the contact rate of CD4^+^ T_RM_ cells with DCs remained unchanged at approximately 20%. Interestingly, the percentage of reactivated CD4^+^ T cells that produced IFN-γ and TNF-α was also approximately 20%, matching the DC contact rate. Since cDC1 and DN cDCs, in addition to some macrophage populations, survived the anti-CSF1R antibody treatment, their roles in CD4^+^ TRM cell survival and reactivation need to be evaluated using genetically engineered mouse models that allow for DC-specific (73) and macrophage-specific depletion (74). In addition, it may be necessary to consider the potential involvement of immune cells other than DCs and macrophages as well as stromal cells, some of which express MHC-II (75, 76), in supporting the survival and function of CD4^+^ T_RM_ cells.

Consistent with previous findings (28, 77), the present study showed that the maintenance of epidermal CD8^+^ T_RM_ cells did not require LCs. However, although LCs were reported to be essential for CD8^+^ T_RM_ reactivation during viral infection (27), the results obtained herein demonstrate that they were dispensable for reactivation in CHS, suggesting distinct reactivation mechanisms between viral infection and CHS.

In summary, the present results indicate that local immune memory mediated by CD4^+^ and CD8^+^ T_RM_ cells in the skin is maintained and functions independently of CSF1R-dependent myeloid cells. Further studies are required to elucidate the mechanisms underlying long-term local immune memory mediated by skin T_RM_ cells.

## Data Availability

The original contributions presented in the study are included in the article/**Supplementary Material**. Further inquiries can be directed to the corresponding author.
