# Supplementary material for: CD4+ skin resident memory T cells preferentially colocalize with dermal Folr2hi macrophages in contact hypersensitivity
Source: Front Immunol. 2025 Jul 28;16:1590687. doi: 10.3389/fimmu.2025.1590687 (PMC12336041; doi:10.3389/fimmu.2025.1590687)
Supplement: Supplementary file 1 [file DataSheet1.pdf]

Supplemental Figure 1

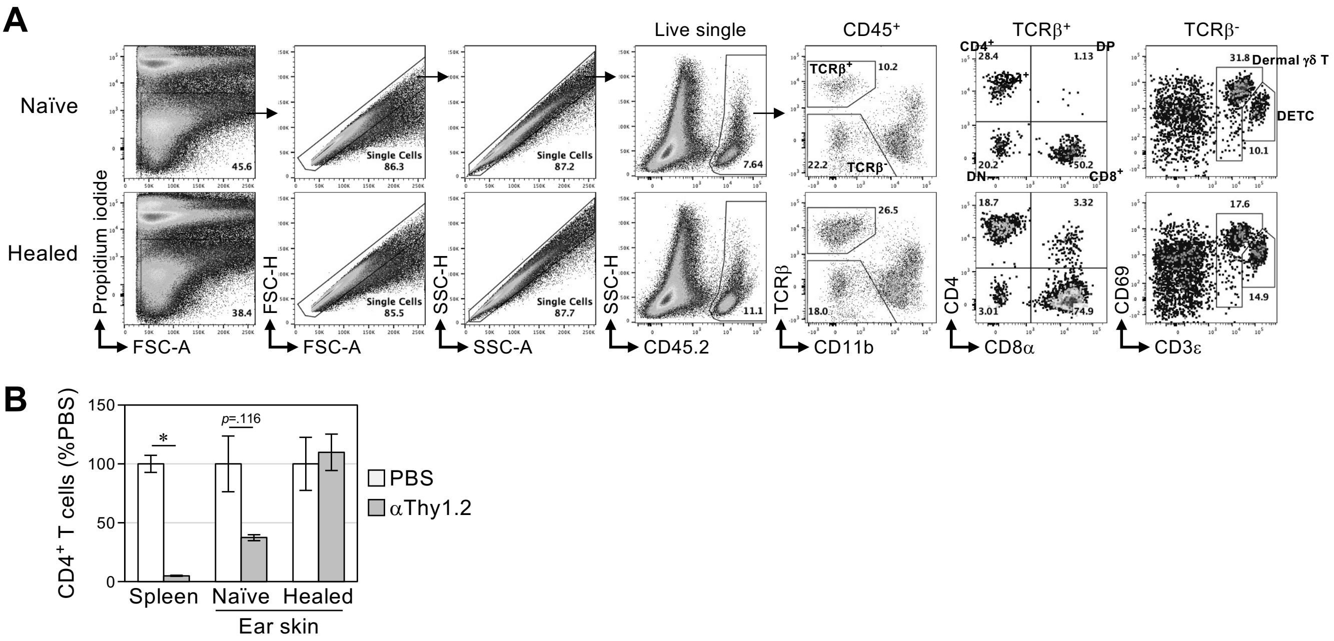

Figure S1. Data related Figure 1.
(A) Flow cytometric gating of ear skin CD4$^+$/CD8$^+$ T_{RM} cells (TCRβ$^+$) and γδ T cells (TCRβ$^-$). DP: double positive. DN: double negative. DETC: dendritic epidermal γδ T cells. **(B)** CD4$^+$ T cells in healed skin, but not naïve skin, were resistant to antibody-mediated cell depletion. After sensitization (day -7) and a challenge (day 0) with TNCB in the right ears, BALB/c mice were injected intraperitoneally with 97 μg of the anti-Thy1.2 (clone: 30H12) antibody on days 44, 46, 48, and 50 to deplete circulating T cells. Control mice were injected with the same amount of PBS. On day 51, cells isolated from the spleen and naïve (left) and healed (right) ears were analyzed by flow cytometry. Spleen data were presented in a previous study (Murata et al., 2020), but are republished for comparison. Data represent the number of CD4$^+$ T cells (CD4$^+$CD3$^+$) per spleen or per ear and are presented as the mean ± S.E. (n = 3, for each). *$P < 0.05$ (two-tailed unpaired *t*-test).

Supplemental Figure 2

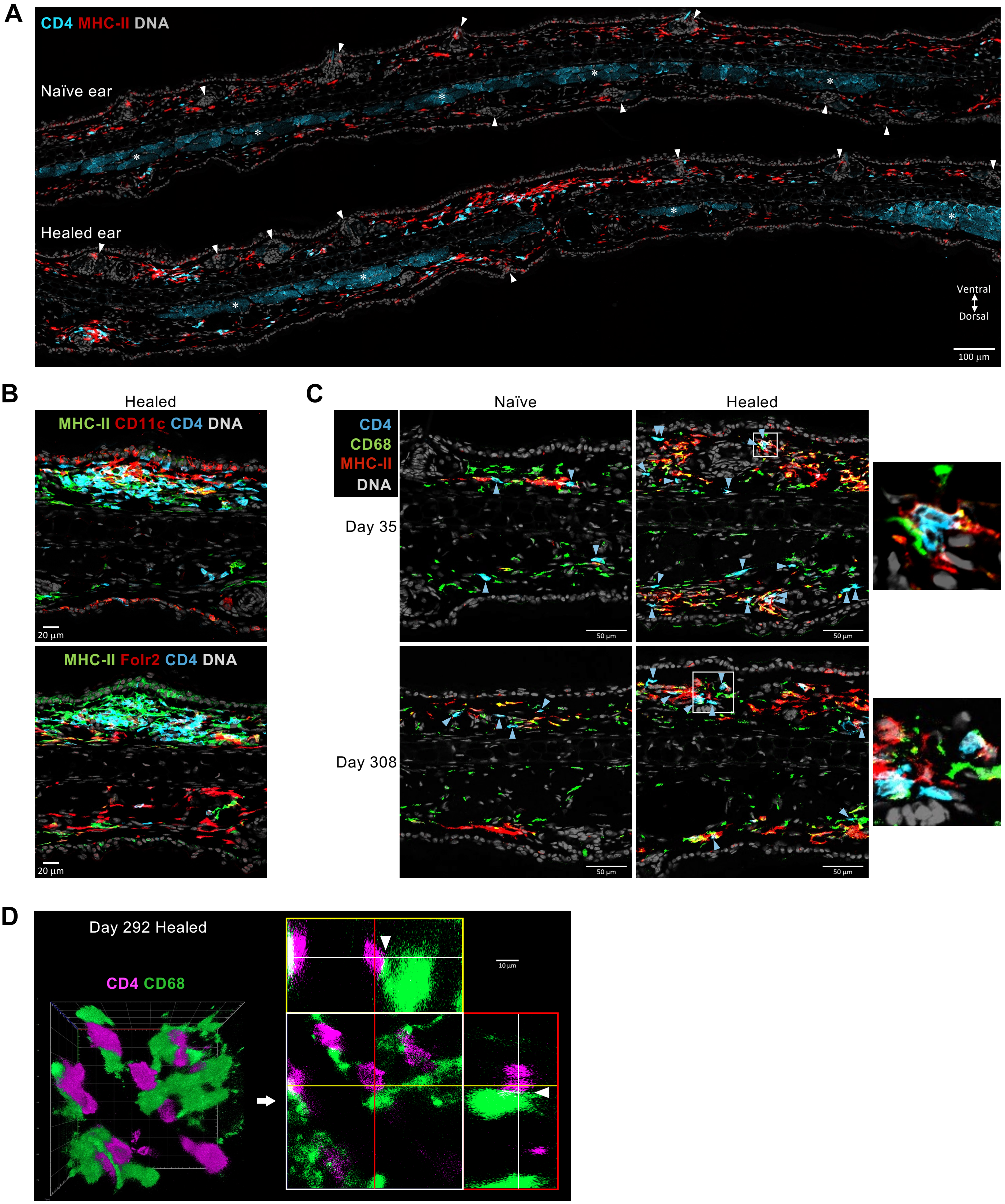

Figure S2. Figures related to Figure 2.
(A) Naïve and healed ear sections on day 308 were stained as in Figure 2B. Arrowheads and asterisks denote hair follicles and the non-specific background fluorescence of muscle layers, respectively. **(B)** An example of a rare cluster of $CD4^+$ T cells and APCs in healed skin. Healed ear skin sections on day 35 were stained with the indicated markers. The cluster contained $CD11c^{+/-}$$MHC\text{-}II^+$ APCs, but few $Folr2^+$ macrophages. **(C)** Representative images of naïve and healed ear skin sections on days 35 and 292 stained with CD4, CD68, and MHC-II. Arrowheads denote $CD4^+$ T cells. **(D)** A dorsal ear half of a healed ear on day 292 was stained with CD4 and CD68. (Left) A z-stack image (thickness: 41.5 µm) of dermal $CD4^+$ T cell-$CD68^+$ myeloid cell interactions. (Right) Orthogonal projections of indicated planes confirmed contact between $CD4^+$ and $CD68^+$ cells (arrowheads).

Supplemental Figure 3

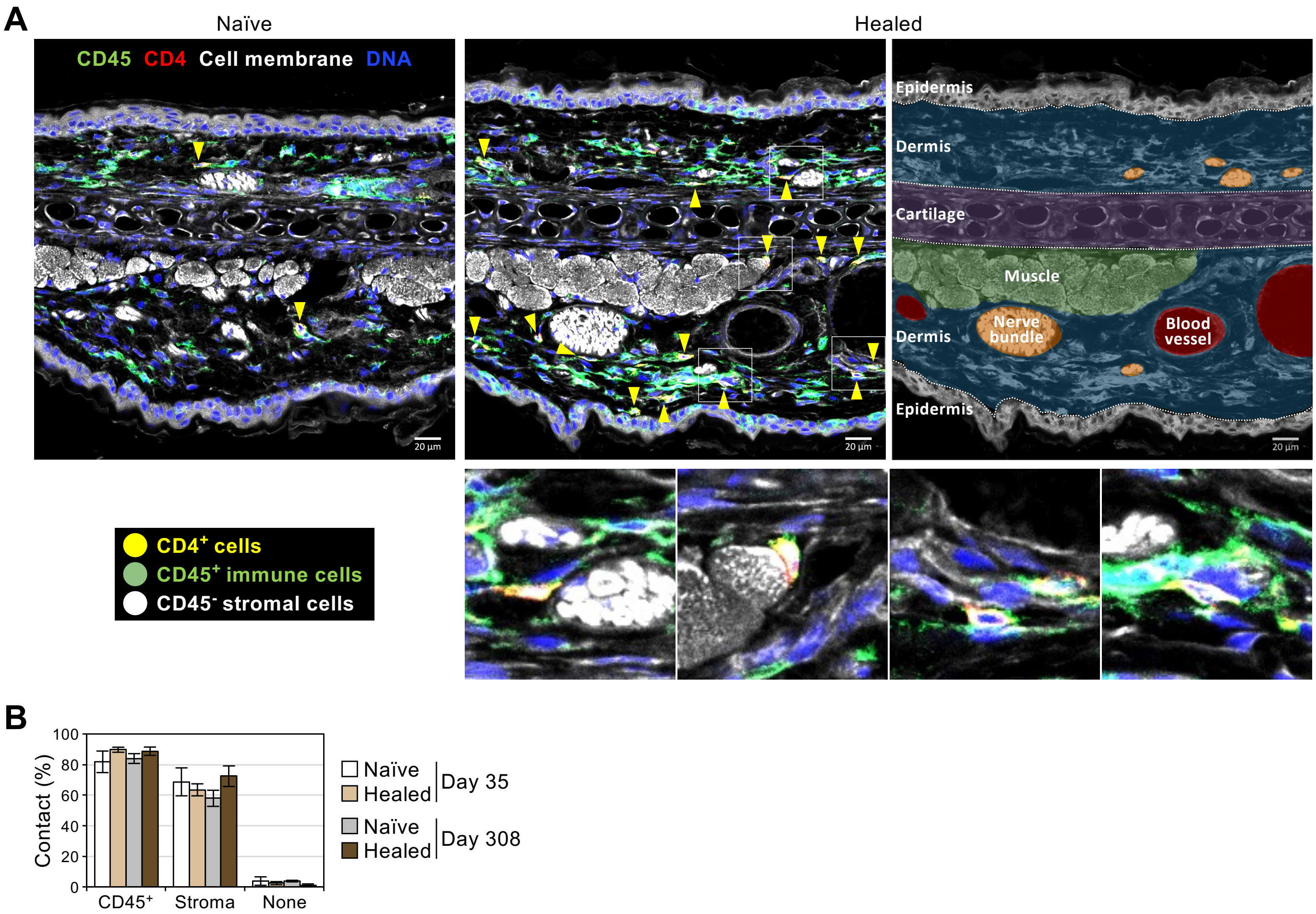

Figure S3. Dermal CD4⁺ T cells are in contact with diverse stromal cells.
Naïve and healed ear sections on days 35 and 308 were stained with the indicated markers. The cell membrane and DNA were stained with CellMask deep red and DAPI, respectively. **(A)** Representative images on day 35 are shown. Arrowheads denote $CD4^+$ cells. The structure of healed ear skin is shown on the right. **(B)** The percentages of scattered $CD4^+$ cell in contact with $CD45^+$ immune cells and $CD45^-$ stromal cells in which only the cell membrane was stained. Data represent the mean ± S.E. (n = 6 for day 35, n = 5 for day 308).

Supplemental Figure 4

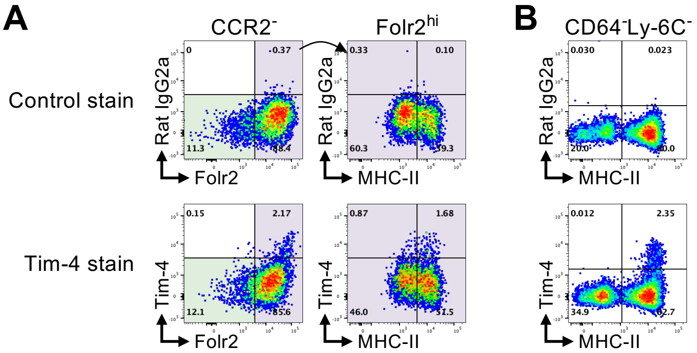

Figure S4. Data related to Figure 4.
(A) Isotype control (rat IgG2a) staining of Tim-4 expression in CCR2$^-$ and Folr2^{hi} populations in healed skin cells, as in Figure 4H. **(B)** Since a subset of dermal cDCs is known to express Tim-4 (48), Tim-4 expression in the MHC-II$^+$ population of the CD45$^+$Lin$^-$CD64$^-$Ly-6C$^-$ fraction containing LCs and cDCs (as in Figure 3B) was also analyzed as a positive control.
